# New Challenges in Tumor Mutation Heterogeneity in Advanced Ovarian Cancer by a Targeted Next-Generation Sequencing (NGS) Approach

**DOI:** 10.3390/cells8060584

**Published:** 2019-06-14

**Authors:** Marica Garziera, Rossana Roncato, Marcella Montico, Elena De Mattia, Sara Gagno, Elena Poletto, Simona Scalone, Vincenzo Canzonieri, Giorgio Giorda, Roberto Sorio, Erika Cecchin, Giuseppe Toffoli

**Affiliations:** 1 Experimental and Clinical Pharmacology Unit, Centro di Riferimento Oncologico (CRO), IRCCS, 33081 Aviano, Italy; rroncato@cro.it (R.R.); edemattia@cro.it (E.D.M.); sgagno@cro.it (S.G.); ececchin@cro.it (E.C.); gtoffoli@cro.it (G.T.); 2 Scientific Directorate, Centro di Riferimento Oncologico (CRO), IRCCS, 33081 Aviano, Italy; marcella.montico@cro.it; 3 Medical Oncology, “Santa Maria della Misericordia” University Hospital, ASUIUD, 33100 Udine, Italy; polettoelena@libero.it; 4 Medical Oncology Unit C, Centro di Riferimento Oncologico (CRO), IRCCS, 33081 Aviano, Italy; sscalone@cro.it (S.S.); rsorio@cro.it (R.S.); 5 Pathology Unit, Centro di Riferimento Oncologico (CRO), IRCCS, 33081 Aviano, Italy; vcanzonieri@cro.it; 6 Department of Medical, Surgical and Health Sciences, University of Trieste, 34127 Trieste, Italy; 7 Gynecological Oncology Unit, Centro di Riferimento Oncologico (CRO), IRCCS, 33081 Aviano, Italy; ggiorda@cro.it

**Keywords:** advanced ovarian cancer, HGSOC, NGS, tumor profile, concurrent somatic mutations, *TP53*, platinum sensitivity, driver actionable genes, translational medicine

## Abstract

Next-generation sequencing (NGS) technology has advanced knowledge of the genomic landscape of ovarian cancer, leading to an innovative molecular classification of the disease. However, patient survival and response to platinum-based treatments are still not predictable based on the tumor genetic profile. This retrospective study characterized the repertoire of somatic mutations in advanced ovarian cancer to identify tumor genetic markers predictive of platinum chemo-resistance and prognosis. Using targeted NGS, 79 primary advanced (III–IV stage, tumor grade G2-3) ovarian cancer tumors, including 64 high-grade serous ovarian cancers (HGSOCs), were screened with a 26 cancer-genes panel. Patients, enrolled between 1995 and 2011, underwent primary debulking surgery (PDS) with optimal residual disease (RD < 1 cm) and platinum-based chemotherapy as first-line treatment. We found a heterogeneous mutational landscape in some uncommon ovarian histotypes and in HGSOC tumor samples with relevance in predicting platinum sensitivity. In particular, we identified a poor prognostic signature in patients with HGSOC harboring concurrent mutations in two driver actionable genes of the panel. The tumor heterogeneity described, sheds light on the translational potential of targeted NGS approach for the identification of subgroups of patients with distinct therapeutic vulnerabilities, that are modulated by the specific mutational profile expressed by the ovarian tumor.

## 1. Introduction

Epithelial ovarian cancer is still the deadliest form of gynecological malignancy. With approximately 295,400 new cases and 184,000 deaths in 2018, ovarian cancer represents the ninth most common form of cancer and the eighth leading cause of cancer-related death among women worldwide [1,2]. Most newly diagnosed women (70–85%) present at an advanced Fédération Internationale de Ginécologie et d’Obstetrique (FIGO) stage III–IV of the disease [3]. Traditionally, management is primary debulking surgery (PDS) followed by first-line platinum-based chemotherapy, usually with a doublet of platinum derivative and taxane [4]. The main prognostic factors identified for clinical outcome in patients with advanced ovarian cancer are tumor stage and mostly residual disease (RD) after PDS, with the goal of surgery being no visible tumor residue (RD = 0) [5,6]. Epithelial ovarian tumors are considered highly heterogeneous with different histological subtypes; based on pathological and molecular characteristics, these tumors have been grouped in type I or II [7,8]. Type I (5–10%) includes low-grade serous, mucinous, clear cell, and endometrioid ovarian carcinomas, are typically *KRAS*, *PIK3CA*, *PTEN*, or *BRAF* mutated, and diagnosed frequently at early stages. Type II (80–90%) includes undifferentiated carcinomas, carcinosarcomas, and the most common type of ovarian cancer, high-grade serous ovarian cancer (HGSOC), which is characterized by poor prognosis and high frequency of *TP53* mutations. As tumor grades G2 and G3 are not substantially different clinically and biologically, are currently used to classify HGSOC for assessment of nuclear atypia [9,10,11]. Heritable mutations in *BRCA1/2*, especially *BRCA1*, increase the risk of developing ovarian and breast cancers; ~20% of HGSOCs are also mutated in the *BRCA1/2* susceptibility genes due to a combination of germline and somatic mutations [12,13]. Advanced ovarian cancer, particularly HGSOC, is usually sensitive to first-line chemotherapy; however, tumors often become pharmacoresistant despite an initial response to surgical debulking and first-line chemotherapy. Approximately 70% of patients will relapse in the first 3 years [14], with a 5-year survival rate of 30% to 40% in most parts of the world [15]; thus, there is an urgent need to improve ovarian cancer treatment. Recent advances in next-generation sequencing (NGS) has allowed extensive molecular profiling of tumors, improving our knowledge of heterogenous ovarian disease by identifying novel mutations and potential actionable therapeutic targets. In the precision medicine era, personalized therapy for each patient is the most attractive challenge, with a great impact on the management of ovarian tumors, but it cannot be approached without genomic knowledge of the tumor to be treated [16]. Identifying the mechanisms involved in the response or resistance to treatment is essential to define tumor profiles when devising precision treatment plans, and future strategies will likely rely on multiple clinical, immunogenomic, and pharmacogenomic factors [17,18]. The aim of the present study was to profile the somatic mutation spectrum of 79 chemo-naïve tumors, including 64 HGSOCs, of patients with advanced ovarian cancer (III–IV stage, tumor grade G2-3) and optimal residual disease (RD < 1 cm) after PDS. Tumor and matched blood samples were selected from a retrospective collection of consecutive ovarian cancer patients who received platinum-based chemotherapy after surgery to identify new genetic markers of platinum-resistance and patient prognosis. Molecular profiles were obtained using a targeted NGS approach with a commercial panel covering 82 exons (all 11 exons of *TP53*) and the related intronic boundaries of 26 cancer-related genes.

The presence of concurrent somatic mutations in two driver actionable genes identified a poor prognostic signature in patients with HGSOC that could be redirected to personalized or investigational treatments. Furthermore, in HGSOC, we observed an association between gain-of-function (GOF) mutations in *TP53* and patient sensitivity to platinum-based treatment.

## 2. Materials and Methods

### 2.1. Patients, BRCA1/2 Testing and Human Ethics

Tumor and matched blood samples were selected from a retrospective collection of consecutive ovarian cancer patients who underwent PDS at CRO-Aviano between 1995 and 2011 before receiving any chemotherapeutic treatment. Tumor staging and grading were assessed according to International Federation of Gynecology and Obstetrics (FIGO) and World Health Organization (WHO) criteria, respectively. Patients included in this study received a diagnosis of advanced ovarian cancer with high grade (G2-G3) and high FIGO stage (III-IV) and were treated primarily with PDS and had an optimal tumor residue (no evident residual disease or residual tumor < 10 mm). Clinico-pathological characteristics, treatment, and complete follow-up information were collected from medical records as current clinical surveillance procedures. At the time of the enrollment (1995–2011), clinical genetic testing of germline mutations in *BRCA1/2* genes was performed routinely only in patients with a documented family history of breast and/or ovarian cancer. We retrospectively retrieved data concerning the *BRCA1/2* germline mutational status from the medical records, when reported. Written informed consent was obtained from each patient with histologically confirmed epithelial ovarian cancer for the use of peripheral blood, tissue samples, and clinical data for research purposes. The study was conducted in accordance with the Declaration of Helsinki and was approved by the Ethics Committee of the CRO Aviano National Cancer Institute, Italy (Institutional Review Board n. CRO-2014-43).

### 2.2. Next-Generation Sequencing Analysis

The Illumina TruSight Tumor 26-genes panel (Illumina, Inc., San Diego, CA, USA; http://www.illumina.com/products/trusight-tumor-26-gene.html) was chosen for NGS analysis. This panel provides coverage of exon coding regions where variation has been cataloged in the COSMIC database in oncogenes, and coverage of all 11 exons and intronic flanking regions of *TP53* tumor suppressor gene. TruSight Tumor 26-genes gives a more comprehensive view of somatic variation in solid tumors, including lung, colon, melanoma, gastric and ovarian cancer. Given the retrospective nature of our study and the possibility to apply this panel in other sample collections from patients with different tumor diseases, TruSight Tumor 26-genes was selected for this type of sequencing approach. Frozen ovarian tumor specimens taken during primary surgery or operative laparoscopy and matched blood samples were analyzed retrospectively. Tumor samples were macrodissected and visually inspected by the pathologist to assess a minimum tumor cellularity of 70%. Genomic DNA was extracted from both the tumor and peripheral blood mononuclear cells (PBMCs) using the EZ1 DNA Tissue Kit and EZ1 DNA Blood 350 µL Kit (Qiagen, Hilden, Germany), respectively, according to the manufacturer’s instructions. DNA samples were quantified using PicoGreen Dye (Quant-iT PicoGreen dsDNA Assay Kit, Thermo Fisher Scientific, Waltham, MA, USA) on a Tecan Infinite 200 PRO reader (Tecan Trading AG, Männedorf, Switzerland) and normalized to 5 ng/μL for successive library preparation. DNA libraries were prepared for NGS according to the manufacturer’s instructions. TruSight Tumor 26-genes (21 Kb in size) screens 82 exons in 26 tumor-related genes (*AKT, ALK, APC, BRAF, CDH1, CTNNB1, EGFR, ERBB2, FBXW7, FGFR2, FOXL2, GNAQ, GNAS, KIT, KRAS, MAP2k1, MET, MSH6, NRAS, PDGFRA, PIK3CA, PTEN, SMAD4, SRC, STK11, TP53*) across 174 amplicons (165–195 bp in size) with 1000× minimum coverage (mean 7000×) for each amplicon. Normalized libraries were analyzed on a MiSeq platform (Illumina) using a V3 (600 cycles) sequencing flow cell with 2 × 121 base-pair analysis set-up. The raw data were automatically processed and analyzed by the Illumina-MiSeq system pipeline. VCF files were imported into the Illumina Variant Studio Data Analysis Software 2.2 for variant calling and imputation. Genetic variants were filtered using the following criteria: PASS filter, variant call quality of 100, frequency of the alternative (Alt) allele ≥ 4% (TruSight Tumor panel achieves limits of detection < 5% variant allele frequency with a minimum cut-off point of 3%), and the total number of reads passing quality filters (read depth) ≥ 1000×. The COSMIC (https://www.cancer.sanger.ac.uk/cosmic), dbSNP (https://www.ncbi.nlm.nih.gov/snp), and ClinVar databases (http://www.ncbi.nlm.nih.gov/clinvar/) and IARC TP53 Mutation Database (http://p53.iarc.fr/ProtocolsandTools.aspx) were searched to determine whether the detected mutations were previously assigned ID numbers.

### 2.3. In Silico Analysis

MutationTaster (http://mutationtaster.org) was used to predict the potential effect of the novel somatic mutations on the structure and function of human p53 protein. PolyPhen2 v2.2.2r398 (http://genetics.bwh.harvard.edu/pph2) and Protein Variation Effect Analyzer (PROVEAN)/SIFT (http://http://provean.jcvi.org/protein_batch_submit.php?species=human) were applied to predict the potential effect of the identified missense mutations. When available, the clinical significance of mutations was assessed with the ClinVar database. Human Splice Finder (HSF 3.1 released on January 10, 2018; http://umd.be/HSF3/index.html) was used to investigate the potential impact of splicing abnormalities caused by the somatic variants in non-consensus splice sites.

### 2.4. Statistical Analysis

Associations between clinico-pathological characteristics and the number of somatic mutations were tested by Fisher’s exact test or the Chi-squared test; to take into account multiple testing, the Bonferroni correction was applied and the *p*-value of 0.05 was divided for the number of genes evaluated in comparisons. To compare continuous variables between two groups, the non-parametric Mann-Whitney test was used. In patients with HGSOC, the effect of somatic mutations on platinum-free interval (PFI), time to recurrence (TTR), or overall survival (OS) was assessed by the Kaplan-Meier method, and the log-rank test was used to test the difference between groups. OS was defined as the interval between PDS and the date of death from any cause or last follow-up. TTR was defined as the interval between PDS and the date of first recurrence/progression or last follow-up. PFI was defined as the interval between the end of the first-line platinum-based treatment and the date of first recurrence/progression or last follow-up. If relapse occurred during treatment or within 4 weeks of the end of platinum treatment, the patient was defined as “refractory”; if the interval was <6 months, the patient was defined as “platinum-resistant”; if the interval was between 6 and 12 months, the patient was “intermediately sensitive”; if the interval was >12 months, the patient was defined as “platinum-sensitive”. Hazard ratios and corresponding 95% CIs were estimated by the Cox proportional hazards model. For all patients, the date of primary surgery can be assimilated to the date of diagnosis. Patients were censored for OS at the date of their last available follow-up visit within 120 months, whereas for PFI and TTR they were censored at the date of their last available follow-up visit within 60 months. Patients lost to follow-up were censored at date of last contact in the analysis of OS, TTR, and PFI.

Multivariable analysis using a Cox regression model was performed to assess other acknowledged prognostic factors potentially associated with patient prognosis. The following variables were considered for inclusion: residual tumor after PDS, FIGO stage, and age at diagnosis. A *p* < 0.05 (two-sided) was considered significant. Due to the small number of patients (n = 15), statistical analyses were not reported for the non-HGSOC subgroup.

## 3. Results

### 3.1. Patient Characteristics

The baseline clinical and demographic characteristics of the 79 patients with advanced ovarian cancer enrolled in this study, including 64 with HGSOC, are summarized in Table 1. The median follow-up was 47.7 months (range, 8.3–190.4 months). Ovarian cancer was diagnosed > 50 years of age in most patients (60/79, 76.0%). Serous was the prevalent tumor histotype. HGSOC patients with platinum-sensitive ovarian cancer were more frequent those with refractory, resistant, and intermediate sensitivity to platinum-based treatment.

All 79 patients underwent PDS, with no visible tumor residue (RD = 0) for 40 patients. PDS was followed by first-line platinum-based chemotherapy, most with carboplatin-paclitaxel regimen. Other treatments were cisplatin-paclitaxel, cisplatin-cyclophosphamide, cisplatin-epirubicin- cyclophosphamide, cisplatin-adriamycin-cyclophosphamide, or not specified (platinum-based). Despite curative treatment, during the observation time, 70 (88.6%) experienced recurrence (56 (87.5%) with HGSOC) and 55 (69.6%) patients died (42 (65.6%) with HGSOC).

### 3.2. Landscape of Somatic Mutations in Advanced Ovarian Cancer

**Figure 1:**
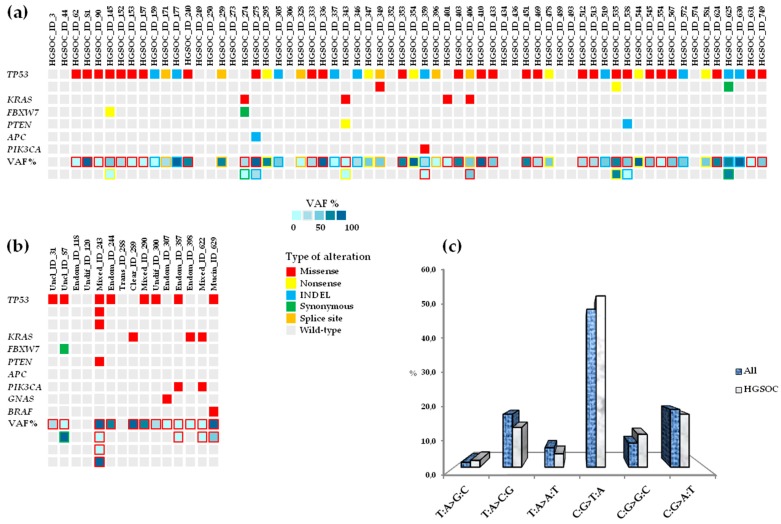
Mutational landscape in advanced ovarian tumors (n = 79) by NGS. (**a**) Somatic profile in HGSOCs (n = 64). (**b**) Somatic profile in non-HGSOCs (n = 15). (**c**) A bar graph represents the single-nucleotide variants (SNVs) found in advanced ovarian tumors. VAF: variant allele frequency; INDEL: insertion or deletion leading to in-frame or frameshift change; HGSOC: high-grade serous ovarian cancer; Uncl: unclassified; Endom: endometrioid; Undif: undifferentiated; Trans: transitional; Clear: clear cells; Mucin: mucinous.

NGS was performed on frozen tissue from 79 patients diagnosed with advanced ovarian cancer and paired blood samples as reference samples. The somatic variants detected were not identified in the paired matched blood samples from each patient. Analysis of the 26 tumor-related genes on the targeted NGS panel was carried out using Variant Studio after filtering genetic variants using the following criteria: PASS filter, variant call quality = 100, frequency of the alternative (Alt) allele ≥ 4%, and total number of reads passing quality filters (read depth) ≥ 1000×. A total of 81 somatic mutations were identified across eight genes (*TP53*, *KRAS*, *FBXW7*, *PTEN*, *APC*, *GNAS*, *PIK3CA*, *BRAF*) in 64 (81%) patients, 52 with HGSOCs (52/64, 81.2%) and 12 out of 15 (80.0%) with non-HGSOC (Figure 1a,b).

**Figure 2:**
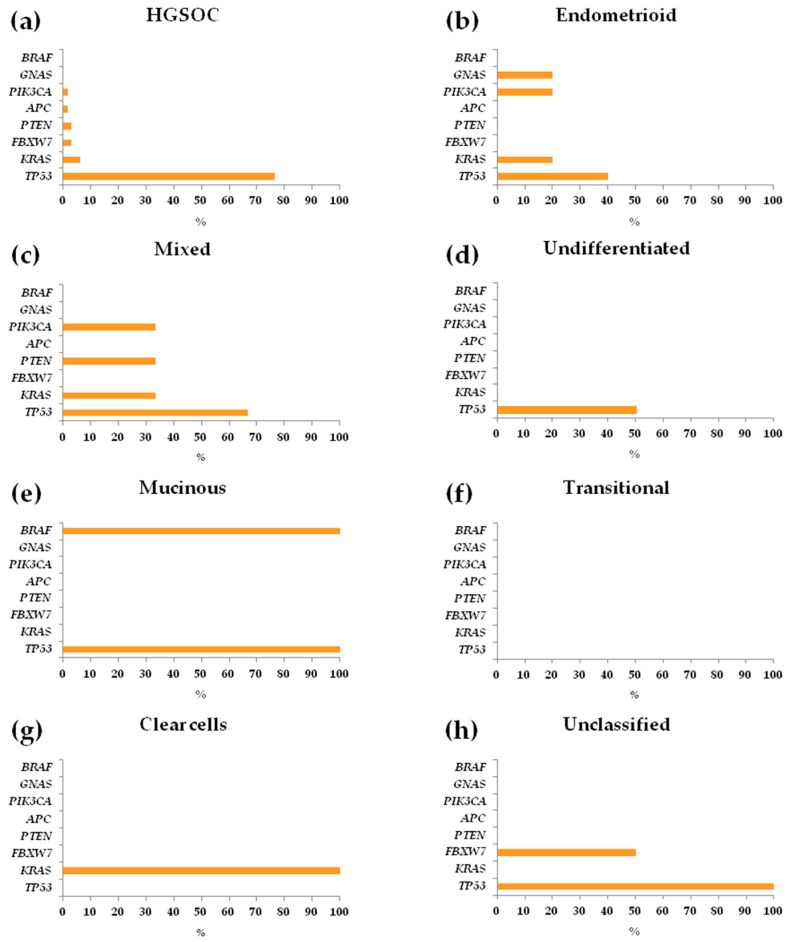
Mutation frequencies by ovarian cancer histological subtypes (n = 79) patients with advanced ovarian cancer. Mutation frequency was calculated as the number of variant occurrences within each gene divided for the total number of patients in the following ovarian cancer histological subtypes: (**a**) HGSOCs; (**b**) Endometrioid; (**c**) Mixed; (**d**) Undifferentiated (**e**) Mucinous; (**f**) Transitional; (**g**) Clear cells; (**h**) Unclassified. HGSOC: high-grade serous ovarian cancer.

For each somatic mutation discovered, ID numbers assigned in the COSMIC database, dbSNP, ClinVar, and International Agency for Research on Cancer (IARC) TP53 Mutation Database (Database R19, released August 2018) [19] were reported when available (Table S1). The variant allele frequency (VAF) ranged from 4.0% to 96.8%, and C:G>T:A transitions were the most frequent type of single nucleotide variants (SNVs), representing 50.0% of changes (54.2% of HGSOC and 38.9% of non-HGSOC samples), followed by C:G>A:T transversions, representing 18.2% of changes (Figure 1c). Mutations were not detected in *AKT1*, *ALK*, *CDH1*, *CTNNB1*, *EGFR*, *ERBB2*, *FGFR2*, *FOXL2*, *GNAQ*, *KIT*, *MAP2K1*, *MET*, *MSH6*, *NRAS*, *PDGFRA*, *SMAD4*, *SRC*, or *STK11* (*BRCA1/2* were not included in the commercial panel). Most patients (57/79, 72.1%) had mutations in *TP53*, followed by *KRAS* (7/79, 8.9%), *FBXW7* (3/79, 3.8%), *PTEN* (3/79, 3.8%), *PIK3CA* (3/79, 3.8%), *APC* (1/79, 1.3%), *GNAS* (1/79, 1.3%), and *BRAF* (1/79, 1.3%). Five additional *TP53* mutations were detected in one patient (#243) with mixed histotype and in three patients (#349, #535, #625) with serous subtype, for a total of 62 variants in this gene. *TP53* mutations occurred within all histological subtypes except transitional and clear cell carcinomas: serous (49/64, 76.6%), endometrioid (2/5, 40.0%), mixed (2/3, 66.7%), undifferentiated (1/2, 50.0%), mucinous (1/1, 100.0%), unclassified (2/2, 100.0%) (Figure 2a–h).

Mutations in *KRAS* were prevalent in mixed (1/3, 33.3%) and endometrioid (1/5, 20.0%) carcinomas, less frequent in serous samples (4/64, 6.2%), and an exclusive event in the clear cell tumor sample (1/1, 100.0%) (Figure 2a–c,g). *FBXW7* mutations were identified in serous (2/64, 3.1%) and unclassified (1/2, 50%) histotypes (Figure 2a,h). *PTEN* mutations were identified in serous (2/64, 3.1%) and mixed (1/3, 33.3%) samples (Figure 2a,c). *PIK3CA* mutations were identified in serous (1/64, 3.1%), endometrioid (1/5, 20.0%), and mixed (1/3, 33.3%) samples (Figure 2a–c). The mutations identified in *APC*, *GNAS,* and *BRAF* were found in serous (1/64, 1.6%), endometrioid (1/5, 20.0%), and mucinous (1/1, 100.0%) tumor samples, respectively (Figure 2a,b,e). The transitional tumor carried no mutations (Figure 2f). Furthermore, the associations between somatic mutations detected (presence vs. absence) and clinico-pathologic characteristics of patients with HGSOC, were evaluated. In HGSOC, mutations were not significantly associated with FIGO stage III or IV, tumor grading (G2 or G3), or tumor residue after PDS (RD = 0 or RD < 1; Table 2), except for platinum-sensitivity: platinum-refractory ovarian cancer was significantly associated with presence of a mutation in *APC* (*p* = 0.0001, Table 2), which was detected in one patient (#275), that had also a concurrent mutation in *TP53* (Figure 1a).

From the medical records, we retrieved data concerning the *BRCA1/2* germline mutational status for four patients. All four patients had a diagnosis of HGSOC; two patients harbored known frameshift mutations in *BRCA1* (#535: *BRCA1* p.E230Gfs*3; #631: *BRCA1* p.K654Sfs*47), whereas *BRCA1* mutations were not described in detail for the two remaining patients (#493, #513). Patient #631 was also a carrier of a silent mutation in *BRCA2* (p.S1733S). Among patients with mutations in *BRCA1*, three were platinum-sensitive (#493, #535, #631) and one exhibited intermediate platinum-sensitivity (#513). Three patients (#513, #535, #631) who were carriers of *BRCA1* mutations also had somatic mutations in *TP53* (Figure 1a).

### 3.3. Repertoire and Distribution of Somatic Mutations in HGSOC and non-HGSOC

HGSOC tumors had a total of 62 somatic mutations: *TP53* (49/64, 76.6%), *KRAS* (4/64, 6.2%), *FBXW7* (2/64, 3.1%), *PTEN* (2/64, 3.1%), *APC* (1/64, 1.6%), *PIK3CA* (1/64, 1.6%) (Figure 2a). The distribution of the main clinico-pathological characteristics, comparing the mutated (n = 52; median age 56 years, range: 31–80 years) and non-mutated (n = 12; median age 58 years, range: 42–81 years) patients to HGSOC, was not significantly different (data not shown). Among the 62 mutations identified, 10 were not reported in the COSMIC database, dbSNP, ClinVar, or the IARC TP53 Mutation Database (Table S1): 7 in *TP53* (p.K320Rfs*11, p.M243_M247del, p.Q52Tfs*66, p.K305*, p.L35S, p.Q331Rfs*14, p.G266_F270del), 1 in *FBXW7* (p.V409V), one in *PTEN* (p.S226Ifs*28), and one in *APC* (p.T1438Kfs*36). Although unreported in public databases, two in-frame INDEL (insertion or deletion leading to in-frame or frameshift change) mutations in *TP53* (p.M243_M247del and p.G266_F270del) were previously validated by our group and aberrant overexpression of nuclear p53 observed in the corresponding tumor samples [20].

Of the newly identified somatic mutations, seven were designated as “disease-causing” and one as “polymorphism” (i.e., probably harmless) in MutationTaster, two “tolerated” in SIFT, one “benign” in Polyphen-2, and two “neutral” in Provean. In addition, six were predicted to potentially or probably affect splicing and two to not have an impact on splicing by HumanSpliceFinder (HSF) (Table S1). Considering the type of alteration in the profiled tumors, HGSOCs had a prevalence for missense mutations (33/62, 53.3%), followed by INDELs (12/62, 19.3%), nonsense (i.e., stop gained) mutations (10/62, 16.1%), splice sites (intronic) in the exon boundaries (6/62, 9.7%), and synonymous mutations (1/62, 1.6%) (Figure S1a).

Moreover, most of the INDELs (10/12, 83.3%) led to frameshifts and, when also considering the nonsense mutations, the number of mutations that cause the translation of truncated proteins was roughly one-third the total amount (20/62, 32.3%). In the subgroup of patients with non-HGSOC, tumors were mutated with a total of 19 somatic mutations: *TP53* (8/15, 53.3%), *KRAS* (3/15, 20.0%), *PIK3CA* (2/15, 13.3%), *FBXW7* (1/15, 6.7%), *PTEN* (1/15, 6.7%), *GNAS* (1/15, 6.7%), and *BRAF* (1/15, 6.7%) (Figure 2b–h).

Among the 19 mutations discovered, two identified in endometrioid ovarian carcinomas were previously unreported (Table S1): p.P110H in *KRAS* and p.C805T in *GNAS*. Both missense mutations were evaluated as “damaging”, “probably damaging”, “deleterious”, “disease-causing” in SIFT, Polyphen-2, Provean, and MutationTaster, respectively, whereas only p.Pro110His was predicted to potentially affect splicing by HSF. In the subgroup with non-HGSOC, INDEL, nonsense, and splice site mutations were not identified; missense alterations were representative of the overall mutational landscape (18/19, 94.7%), except for the presence of a synonymous change (1/19, 5.3%). Patient prognosis was not significantly different between HGSOC patients and non-HGSOC patients with the uncommon ovarian histotypes (data not shown).

### 3.4. Concomitant Mutated Driver Genes in HGSOC and Impact on Clinical Outcome

**Figure 3:**
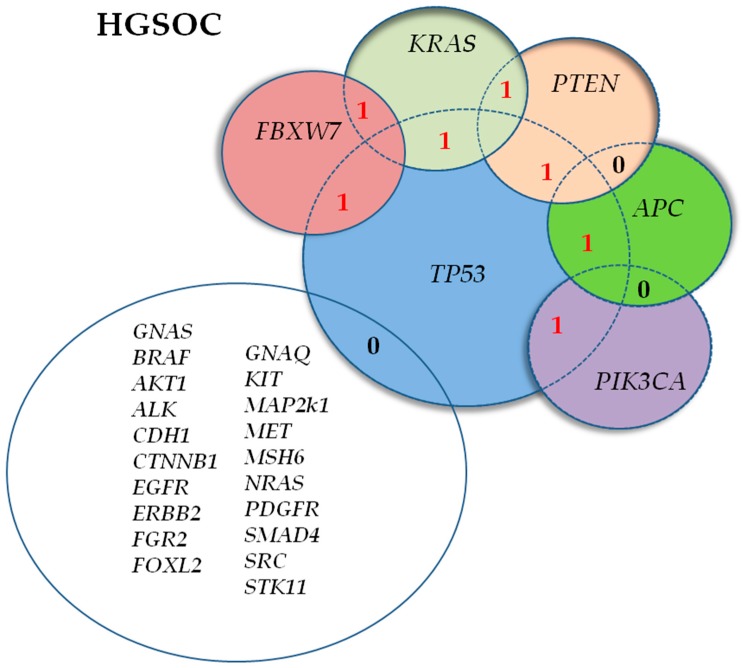
Number of concurrent mutations identified in driver actionable genes of the panel in seven patients with high-grade serous ovarian cancer (HGSOC).

Two concurrent somatic mutations in two different genes were identified in 15.2% (12/79) of advanced ovarian tumors, and 11.0% of patients with HGSOC (7/64): #145, *TP53*(p.R249S)/*FBXW7*(p.R393*); #274, *KRAS*(p.G12D)/*FBXW7*(p.V409V); #275, *TP53*(p.R248Q)/*APC*(p.T1438Kfs*36); #343, *KRAS*(p.G12D)/*PTEN*(p.R233*); #359, *TP53*(p.Q331Rfs*14)/*PIK3CA*(p.H1047L); #406, *TP53*(IVS4+5G>A)/*KRAS*(p.G12C); and #538, *TP53*(p.C275F)/*PTEN*(p.S226Ifs*28) (Figure 3).

Besides the inter-tumor heterogeneity, five out of seven (71.4%) of these HGSOCs showed significant (not shown) intra-tumor molecular heterogeneity: for example, sample #538 had a VAF of 58.9% for mutation identified in *TP53* coexisting with a *PTEN* mutation (see Table S1). Regarding the clinical significance of the 14 mutations predicted by ClinVar, four were unreported and had an unclassified clinical value, two were classified as variants with uncertain significance (VUS), four “pathogenic”, two “pathogenic/likely pathogenic”, and two “likely pathogenic” (Table S1). Tumor samples with more than two simultaneously mutated genes were not identified. PFI was not significantly different in these patients compared to those with one or no (0) mutated genes (Log-rank *p* = 0.139, Figure S2a); however, in both univariate (HR_uni_ = 2.55, 95% confidence interval [CI]: 1.10–5.92, *p* = 0.029) and multivariate (HR_mult_ = 3.10, 95% CI: 1.13–8.48, *p* = 0.028) analyses, the median PFI was significantly shorter in patients with two concurrent somatic mutations detected in two different genes (Table 3).

The same trend in PFI was observed when the subgroup of patients with two mutated genes was compared to all other patients (0 or 1) with HGSOC (Log-rank *p* = 0.056, Figure S2b), with a significant difference in both the univariate (HR_uni_ = 2.17, 95% CI: 1.14–4.11, *p* = 0.018) and multivariate (HR_mult_ = 2.44, 95% CI: 1.19–4.99, *p* = 0.015) analyses (Table 3). Similarly, a worse outcome for HGSOCs with two mutated genes was observed for TTP and OS. TTP was not significantly different among the three subgroups (Log-rank *p* = 0.123, Figure S2c), but in both univariate (HR_uni_ = 2.57, 95% CI: 1.07–6.19, *p* = 0.035) and multivariate (HR_mult_ = 3.14, 95% CI: 1.13–8.74, *p* = 0.028) analyses, patients with two concurrent mutated genes had a significantly shorter TTR (Table 3). When an analysis was performed comparing two subgroups (>1 vs. 0 or 1), the TTR was significantly lower in patients carrying two somatic mutations in two genes compared to all others with HGSOCs (Log-rank *p* = 0.046, Figure S2d) in both the univariate (HR_uni_ = 2.25, 95% CI: 1.19–4.26, *p* = 0.012) and multivariate (HR_mult_ = 2.54, 95% CI: 1.27–5.09, *p* = 0.008) analyses (Table 3). In the univariate analysis, OS was not significantly different between the three subgroups (Log-rank *p* = 0.177, Figure S2e), but in the multivariate (HR_mult_ = 3.40, 95% CI: 1.14–4.11, *p* = 0.018) analysis, patients with two concurrently mutated genes had significantly shorter OS (Table 3). OS was not significantly different between the two subgroups (>1 vs. 0 or 1) (Log-rank *p* = 0.077, Figure S2f), but similar to the other results, significantly shorter OS was observed in patients carrying two somatic mutations in two genes compared to all others with HGSOCs in both the univariate (HR_uni_ = 2.06, 95% CI: 1.19–3.56, *p* = 0.009) and multivariate (HR_mult_ = 2.47, 95% CI: 1.50–4.07, *p* = 0.0001) analyses (Table 3). The distribution of the main clinico-pathological characteristics of patients with HGSOCs stratified according to the tumor mutation profile detected by NGS was analyzed to search for clinical factors that may partially explain differences in the outcomes (Table 4).

No significant differences were found, except for platinum sensitivity, when comparing patients with two synchronous mutations in two genes (>1) and those with one mutated gene or unmutated (>1 vs. 0 or 1, *p* = 0.019) or one mutated gene (>1 vs. 1, *p* = 0.004; Table 4). In particular, in the subgroup with co-occuring somatic alterations, patients with platinum-refractory ovarian cancer were prevalent, whereas patients with platinum-sensitive ovarian cancer were less frequent. Specifically, two patients (#274 and #275) of the three with platinum-refractory ovarian cancer had two simultaneous mutations in two driver genes. In five of the 15 patients with non-HGSOC (#87, unclassified, *TP53*(p.A159D)/*FBXW7*(p.D400D); #243, mixed, *TP53*(p.D208I)/*PTEN*(p.M35V); #387, endometrioid, *TP53*(p.R248Q)/*PIK3CA*(p.R93W); #622, mixed, *KRAS*(p.G12D)/*PIK3CA*(p.H1047R); #629, mucinous, *TP53*(p.C176F)/*BRAF*(p.V600E)), two concurrent somatic mutations in two different genes were identified. These 10 mutations have already been reported in public databases, but for ClinVar, six had an unclassified clinical value: n = 3 “pathogenic”, n = 1 “pathogenic/likely pathogenic” and n = 2 “likely pathogenic” (Table S1). In non-HGSOC subgroup, a correlation beetween patients with two concurrent mutations in two driver genes (n = 5) and a reduced PFI, TTR, and OS compared to the others (n = 10) was observed (not shown).

### 3.5. Somatic Spectrum of TP53 Mutations in Patients with HGSOC and Impact on Clinical Outcome

**Figure 4:**
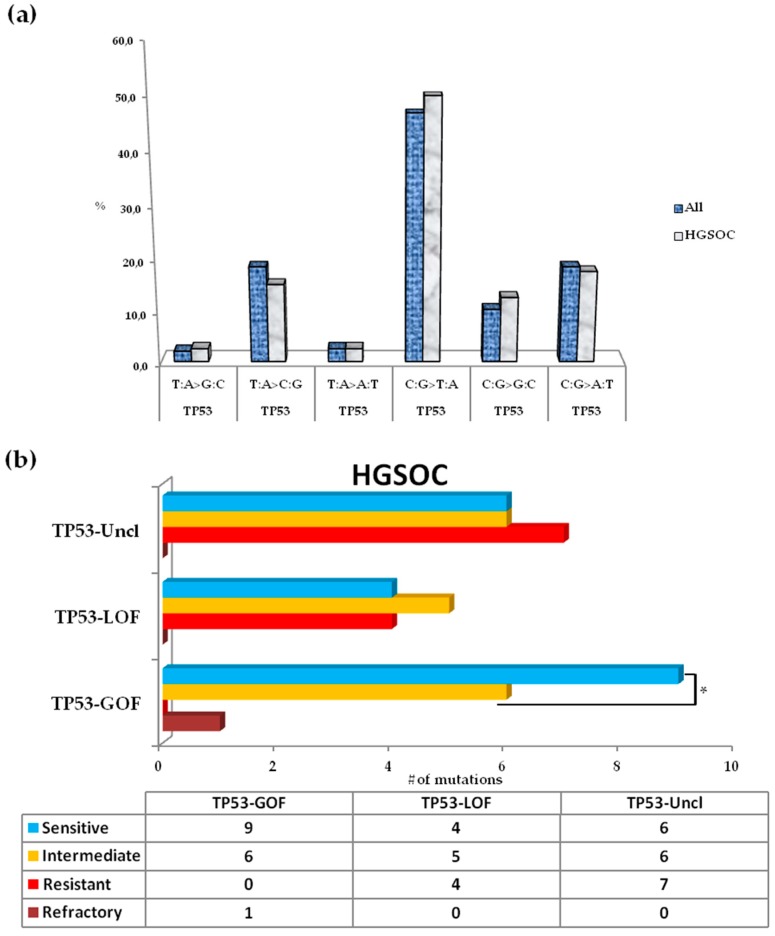
*TP53* mutational landscape and correlation with platinum sensitivity. (**a**) A bar graph represents the SNVs in *TP53* found in advanced ovarian tumors (All, n = 79), including HGSOCs (HGSOC, n = 64). (**b**) Distribution of GOF, LOF and Uncl mutations in *TP53* according to platinum sensitivity in HGSOCs. HGSOC: high-grade serous ovarian cancer. GOF: gain-of-function; LOF: loss-of-function; Uncl: unclassified. *p value < 0.05.

*TP53* was the most frequently altered gene; C:G>T:A transitions were the most abundant (46.9% in all samples, 50% in HGSOC) SNVs, followed by C:G>A:T and T:A>G:C transversions (both 18,4%, Figure 4a). Among SNVs, 15 mutations in *TP53* were located at CpG sites, including 13 in HGSOCs (13/49, 26.5%; Table S1). These 13 mutations at CpG sites were all clustered in p53-DBD. The most common base changes were C:G>T:A transitions (69.2%), followed by C:G>G>C (23.1%) and C:G>A:T (7.7%) transversions. A total of 87.10% of mutations, 82.70% from HGSOC tumors, were mapped in exons 4–8 (codons 102-292) of the DNA binding domain (DBD). The most common *TP53* mutations were identified in DBD in the hotspot residues R273H and Y220C in 3.8% (3/79) of patients, followed by R248Q, R273C, C176Y, R249S, and Q192* in 2.5% (2/79) of patients. In HGSOC tumors, evaluation of the type of *TP53* alteration revealed a similar trend for the overall mutation profile, with prevalent missense mutations (28/52, 53.8%), followed by INDEL (10/52, 19.2%), nonsense (7/52, 13.5%), splice site (6/52, 11.5%), and synonymous (1/52, 1.9%) mutations (Figure S1b). Moreover, most INDELs (8/10, 80.0%) were represented by frameshift mutations; also considering the nonsense variants, approximately one-third of alterations cause the translation of a truncated p53 protein (15/62, 28.8%). INDEL, nonsense, and splice site mutations were not identified in non-HGSOC; all of the alterations were of the missense type (10/10, 100%). Patients carrying at least 1 (range 1–3) somatic variant in *TP53* were compared regarding the clinical outcome of patients without a somatic variant in *TP53*. The *TP53* mutation status was not significantly associated with PFI, TTR, or OS, even when considering patients with HGSOC (Figure S3, Table S2). In HGSOC tumors, mutations in *TP53* were categorized into three main categories by stringent criteria as described previously [21]: GOF (16/51, 31.4%), loss-of-function (LOF; 15/51, 29.4%), and unclassified (20/51, 39.2%). One synonymous mutation (p.Pro89Pro) corresponding to a wild-type mutation that did not alter the *TP53* sequence was found in one patient with platinum resistance. We observed that patients resistant to platinum treatment completely lacked GOF mutations, which were prevalent in patients sensitive to platinum. In patients with platinum resistance, the unclassified variants were the most abundant (7/11, 63.6%), followed by LOF mutations (4/11, 36.4%). In patients with platinum sensitivity, the trend was inverse with GOF mutations being the more frequent type of p53 alteration (9/19, 47.4%), followed by unclassified (6/19, 31.6%) and LOF mutations (4/19, 21.0%). In patients with platinum-intermediate sensitivity, the unclassified and GOF mutations were slightly more prevalent (6/17, 35.3%) with respect to LOF mutations (5/17, 29.4%). In the only patient with a somatic mutation in *TP53* and platinum-refractory HGSOC, a *TP53*-GOF mutation was identified: this patient had also a simultaneous frameshift mutation in *APC* (Figure 1a, Table 2). The distribution of *TP53*-GOF mutations was significantly different (*p* = 0.012, Figure 4b) between platinum-resistant and -sensitive patients. In platinum-sensitive patients the level of sensitivity to platinum was not due to the presence of a companion mutation being GOF mutations prevalently represented by “lonely” mutations in *TP53*: only one patient (1/9, 11%) had another concurrent mutation (*TP53*-GOF/*FBXW7*); moreover, LOF and unclassified were *TP53* solely mutations. Similarly, in platinum-resistant patients LOF mutations had not a companion mutation in another gene of the panel, and among unclassified mutations only one patient (1/7, 14.3%) had a concurrent mutation (*TP53*-Uncl/*PTEN*). Sixty-nine percent (11/16) of *TP53*-GOF mutations in patients with HGSOC involved nucleotide substitutions at CpG sites in the p53-DBD; 72.7% (8/11) were represented by C:G>T:A transitions (Table S1). Improved OS, even if not significant, was observed using the Log-rank test for patients carrying *TP53*-GOF mutations (data not shown).

## 4. Discussion

To identify new genetic markers predictive of platinum resistance and prognosis in patients with advanced ovarian cancer (III–IV stage, tumor grade G2-3) with sub-optimal (RD < 1 cm) tumor residue after PDS, we profiled the somatic mutation spectrum in a retrospective collection of 79 frozen specimens from chemo-naïve tumors, including 64 HGSOCs, using targeted NGS. We report the identification of somatic mutations in clinically actionable and targetable genes [22,23,24] in HGSOC (*TP53*, *KRAS*, *FBXW7*, *PTEN*, *APC*, *PIK3CA*) and non-HGSOC (*TP53*, *KRAS*, *FBXW7*, *PTEN*, *APC*, *PIK3CA*, *BRAF*, *GNAS*) tumors. In the analyzed HGSOC tumor samples, in addition to the most frequently mutated *TP53* gene, aberrations in the other driver genes have been reported in the TCGA dataset in G2-G3 (III–IV stage) serous tumors as provided by Illumina’s Manifest Files [25] and in another studies in HGSOC [26,27] using NGS technology. The dualistic classification of EOC as type I and type II has led to a convenient simplification of the molecular profiles of ovarian tumors [7]. To date, the mutational landscape of non-serous tumors has not been explored extensively as in the serous ovarian tumor subtype. However, recent high-throughput sequencing studies have shown that type I tumors are very heterogeneous and should be considered as different diseases [28]. The molecular profile of non-HGSOC tumors supports this and the current hypothesis that mutations in *TP53* are not a driver event for only type II-HGSOCs, leading to future refinement of EOC classification [28,29]. For example, endometrioid ovarian tumors are characterized by mutations in *ARID1A* and *PIK3CA*, whereas mutations in *TP53* are considered a very uncommon event. Furthermore, mutations in *KRAS* and *BRAF* have been described as rare alterations in G1-G2 tumors [30,31]. Among the five endometrioid ovarian tumors in our series, we detected somatic mutations in *TP53* (2/5), *PIK3CA* (1/5), *GNAS* (1/5), and *KRAS* (1/5) in a G3 tumor. Mutations in *GNAS* have been documented in mucinous [32], clear cell, and serous [27] advanced ovarian cancers; thus, our findings in the endometrioid ovarian cancer subtype should be considered as a very infrequent somatic mutational event. Notably, in HGSOC, all alterations in *KRAS* were clustered in the hotspot codon 12, and 75% (3/4) of them codify for the G to D change. In one patient with HGSOC, the *KRAS* p.G12C mutation in the same hotspot codon was concomitant with an intronic *TP53* mutation in the exon 4 boundaries (IVS4+5G>A). Both mutations (p.G12D/C) affecting codon 12 in *KRAS* have been reported to be poor prognostic markers [33,34,35]. In addition, synchronous mutations in *TP53* and *KRAS* have been reported to be prevalent in low-grade serous ovarian carcinomas, but have been also identified in HGSOC [25,36,37]. The activating hotspot mutation G12V in the pivotal oncogene *KRAS* has been described as the most common single somatic molecular alteration in mucinous ovarian tumors [7,27,38]. We detected *KRAS* p.G12V in the only clear cell tumor among our samples. However, *KRAS* p.G12V was also described recently in ovarian clear cell carcinoma via whole exome sequencing [39], though this subtype is mainly characterized by frequent activating mutations in *PIK3CA* and infrequent mutations in *KRAS* [40].

An interesting observation considering the overall aberrations found in mutated genes evaluated here is the relative abundance of C:G>A:T transversions (18.4%). In colorectal cancer, the frequency reported for this type of transversion was 8.2% [41]. Such SNVs have been related to exposure of tissues or organs to mutagenic agents, particularly tobacco smoking in lung cancer and aflatoxins in liver cancer [41,42]. Thus, the dietary habits and particular life styles of the patients could play a relevant role in the oncogenic process.

The mutation pattern of profiled tumors may have prognostic implications. The application of modern sequencing technologies is leading to a rethinking of the concept of occurrence of lonely driver mutations as stand-alone factors [43]. In the last few years, a tumor genetic signature defined by two or more concurrently mutated genes was correlated with worse patient prognosis and response to treatment in different types of tumors [44,45,46,47,48,49]. These studies highlighted how concomitant mutations can occur within the same tumor, and their interaction may influence sensitivity to anticancer drugs and the final response to chemotherapy, as well as survival. We identified a poor prognostic signature in 11% of patients with HGSOC and harboring concomitant mutations in two driver actionable genes: *TP53*/*FBXW7*, *TP53*/*APC*, *TP53*/*PIK3CA*, *TP53*/*KRAS*, *TP53*/*PTEN*, *KRAS*/*FBXW7*, or *KRAS*/*PTEN*. To the best of our knowledge this is the first study reporting an unfavorable outcome in patients with advanced HGSOC whose tumors showed the described inter- and intra-tumor heterogeneity in molecular profiles. Concomitant mutations in two driver druggable genes were also identified in five non-HGSOC tumors (*TP53*/*FBXW7*, *TP53*/*PTEN*, *TP53*/*PIK3CA*, *KRAS*/*PIK3CA*, *TP53*/*BRAF*), however, the sample size was too small to assess statistical significant conclusions. HGSOC and synchronous mutations in actionable/driver genes was significantly associated with being unresponsive to platinum-based treatment. Specifically, two out of three patients with refractory HGSOC, had concomitant *KRAS*/*FBXW7* and *TP53/APC* mutations.

Inactivating mutations in *FBXW7* leading to LOF sensitizes cells to mTOR inhibitors; mTOR is one of the substrates of *FBXW7*-mediated protein degradation [50]. Moreover, *APC* is a downstream substrate of the FBXW7/cyclinE signaling pathway [51]. In FBXW7-deficient cells, cyclinE expression is increased, leading to final inactivation of APC [52]. Furthermore, FBXW7-mediated cyclinE degradation can be inhibited by an activating mutation in *KRAS* [53]. Recent NGS screening in different types of advanced tumors, including ovarian cancer, highlighted the presence of *FBXW7* mutations that occurred in isolation (12%) or, more frequently (88%), with a concomitant aberration in one or two genes [46]. The prevalent simultaneously mutated gene was *TP53*, as identified in the only ovarian serous tumor sample also mutated in *FBXW7* and *PIK3CA* (1/40, 2.5%); most of the colorectal tumors (86%) analyzed that were positive for mutations in *FBXW7* had a concomitant mutation in *KRAS* [46]. In this study, the limited therapeutic efficacy in patients with concomitant mutations in *FBXW7* and one or two genes who were treated with mTOR inhibitors was supposed to be dependent on the different contributions of the simultaneous aberrations [46]. *TP53*-mutated tumors were also reported to induce epigenetic silencing of *FBXW7* expression with enhancement of the malignant ovarian tumor phenotype [54]. One of the seven patients with HGSOC and poor prognosis had a concomitant *TP53* GOF mutation (p.G249S) and LOF mutation in *FBXW7* (p.R393*).

Of the seven HGSOC tumors characterized by a poor prognostic signature, two had mutations in *KRAS* and *TP53* with concomitant LOF mutations in *PTEN*: *PTEN*(p.R233*)/*KRAS*(p.G12D) and *PTEN*(p.S266Ifs*28)/*TP53*(p.P275F). The patient mutated in *PTEN*/*KRAS* was platinum-sensititive while the patient mutated in *PTEN*/*TP53* was platinum-resistant. Alterations in genes of the PIK3CA/AKT/mTOR signaling network (e.g., *PIK3CA* and *PTEN*), a pathway regulating many biological processes, including cell survival, proliferation, tumorigenesis, metastasis, and resistance to chemotherapy, have been identified in type I and II tumors but are relatively more common in non-HGSOCs [40,55]. Perturbation of the PI3KCA-mediated pathway (acquisition of somatic GOF mutations within *PIK3CA*), results in increased AKT-dependent or AKT-independent signaling; the pathway is antagonized by the activity of the PTEN phosphatase [56]. In particular, loss of PTEN activity resulting from mutations or gene deletions are the most common indirect mechanisms of *PI3KCA* activation in tumorigenesis [57]. Preclinical models and early clinical data have suggested that *PIK3CA* and *PTEN* mutations may predict sensitivity to treatment with PI3K/AKT/mTOR inhibitors, such as everolimus, in multiple tumor types, including high-grade epithelial ovarian cancer [50,58]. Everolimus is under investigation in combination with letrozole in clinical studies of ovarian cancer and endometrial cancer [59,60], and also in combination with bevacizumab [61]. Recent findings in breast cancer, in which the mTOR/Akt/PI3K axis is the most frequently enhanced oncogenic pathway, have suggested a possible synergy for ovarian cancer patients between PI3K inhibitors and poly-adenylate ribose polymerase (PARP) inhibitors. There are ongoing clinical studies in both breast and ovarian carcinoma to assess the efficacy of different combinations of PI3K inhibitors and olaparib [62,63].

The *PIK3CA* mutations p.H1047R (exon 20) and p.R93W (exon 1) are both located in the catalytic p110α subunit of the PIK3α heterodimeric protein [64]. Sequencing of *PIK3CA* in different types of tumors revealed that ~80% of all *PIK3CA* mutations occurred within exons 9 (E542K or E545K) and 20 (H1047R or H1047L), which encode the C-terminal helical and kinase domains of p110α; the rest of the mutations (~20%) were within exons 1–7, which encode the N-terminal domains of p110α, including the p85/adaptor-binding domain (ABD) where p.Arg93Trp is localized [57]. In endometrial carcinoma, codon R93 is frequently mutated, suggesting its investigation in view of direct targeted therapies [52]. *PIK3CA* p.H1047R is considered a hotspot/GOF mutation with known transforming capacity [64]. Beyond the demonstration of strong potential for driving tumor development in the preclinical setting, *PIK3CA* p.H1047R has shown sensitivity to the mTOR inhibitor everolimus [57,65]. In patients with different types of advanced cancers, including ovarian, the simultaneous presence of mutations in *PIK3CA* (especially p.H1047R) and *KRAS* in codons 12 or 13, has been associated with resistance to therapy with PIK3CA/AKT/mTOR inhibitors, although data were not completely confirmed in multivariate models [44]. In other recent studies performed on patients with stage II-III colorectal cancer [45] and early breast cancer [49] who received 5-fluorouracil-based and anthracycline-taxanes adjuvant chemotherapy respectively, concomitant presence of *PIK3CA*-*TP53* mutations was a significantly poor predictive factor for OS [45] and DFS [49], also in adjusted analyses. Notably, the most frequent combinations detected in these patients included *PIK3CA*(p.Met1004Ile)/*TP53*(p.R248Q) and *PIK3CA*(p.H1047R)/*TP53*(p.R273H) [44]. Remarkably, among the seven HGSOC patients characterized by poor prognosis, one had a simultaneous mutation in *TP53* (p.Q331Rfs*14) and *PIK3CA* (p.H1047R).

We also identified concomitant *BRAF/TP53* mutations in the only mucinous ovarian cancer sample, particularly the activating mutation p.V600E in *BRAF* and missense change p.C176F in *TP53*. Mucinous ovarian carcinomas are frequently mutated in *KRAS* and to a lesser extent *TP53*, and recent studies using NGS technology revealed activating mutations in *BRAF* (5–23%) [32,66], demonstrating frequent RAS-RAF-MEK-ERK pathway activation. The concomitant combination *BRAF*(p.V600E)/*TP53* is rare and has only been described recently in mucinous ovarian cancer [32]. In melanoma, colorectal, and lung cancer, *BRAF* p.V600E is a well-known druggable (anti-EGFR therapy) hotspot affecting the kinase domain of the protein, recently classified as a class I mutant, with the strongest kinase activity, constitutive MAPK cascade, and RAS-independent [67,68].

In our study, *TP53* was the most frequently mutated gene in patients with another mutated actionable gene, in both HGSOCs (5/7) and other uncommon ovarian histotypes (4/5). This result is not surprising or unexpected, as *TP53* is the predominant mutated gene in epithelial ovarian cancer [69] and in our series. Furthermore, 26% of all *TP53* mutations identified in HGSOC were represented by single-base missense substitutions located at CpG sites in the functional DBD; most of them (69%) were *TP53* GOF mutations, most due to C:G>A:T transitions (72.7%). Methylated cytosines at CpG dinucleotides are less stable and undergo spontaneous deamination into thymine at a rate 10-times higher than other nucleotides [70]; thus, an estimated one-third of transition mutations are responsible for the onset of human cancers and genetic diseases. However, other mechanisms have been related to a higher frequency of substitution at CpG sites, such as exogenous carcinogens (e.g., benzo(a)pyrene or UV/sunlight) having greater affinity for methylated CpG dinucleotides [71]. Besides the mutations in p53 have been largely associated to transformation [72], recent studies have focused on a further stratification of p53 variants in view of their oncomorphic potential [21] Nonetheless, in patients with HGSOC, we observed an inverse association between GOF mutations and platinum-resistance (tumors with *TP53*-GOF mutations were more sensitive to platinum-based treatment) with respect to what was reported for TCGA (tumors with *TP53*-GOF mutations were more resistant to platinum-based treatment) [21,73]. Intriguingly, in tumors from patients with platinum-resistant ovarian cancer, *TP53*-GOF mutations were not detected, but we found the highest rate of unclassified *TP53* mutations, including missense and splice site mutations with uncertain phenotypic effects. From medical records we retrieved data about germline *BRCA1* mutations that were reported in four patients with HGSOC who shared a better response to platinum-based treatment and a favorable prognosis (median OS >73 months) [12,74]. These patients with platinum-sensitive recurrent HGSOC and mutations in *BRCA1*, could have benefited of maintenance therapeutic approach with PARP inhibitors, but at the time of the enrollment these pharmacological agents were not applied in clinical practice. Beyond *BRCA* alterations, mutations in other genes involved in the homologous recombination (HR) pathway respond to PARP inhibitors therapy; further, alterations in upstream HR modulators such as in *PTEN*, may induce also a “BRCAness” phenotype that would potentially increase tumor sensitivity to PARP [75]. Notably, the patient with a frameshift mutation in *PTEN* (and a simultaneous mutation on *KRAS*), was a platinum-sensitive recurrent HGSOC that could had been a candidate for PARP inhibitors based-therapy. Frameshift and nonsense mutations encoding for truncated proteins due to the formation of a premature stop codon represent approximately one-third of all gene variations from the 26 cancer-gene panel found in HGSOC samples. The major contribution in truncating variants was due to the *TP53* mutational spectrum (*TP53*-LOF mutations). Conversely, alterations in non-HGSOC were all localized in the DBD and associated with the translation of full-length monomers. These data could be suggestive of a strong selective pressure to maintain the expression of untruncated monomers with increased stability of p53 mutants [72,76]. Our observation should be affected by the small number of uncommon subtypes investigated here. Nevertheless, the data are concordant with recent genomic profiling studies in mucinous and clear cell ovarian carcinomas in which targeted NGS was applied [39,77,78]. Overall, our results for the characterization of *TP53*-GOF/LOF/unclassified mutations may improve the development of cancer-specific drug targets, with p53 as an attractive druggable target [79,80]. In particular, the direct targeting of missense mutant p53 proteins (mutp53) at preclinical and clinical levels, and in association with other anti-cancer therapies, is of increasing interest, even if targeted drugs are still at the early stages of development and clinical implementation progressing slowly [81,82,83].

In conclusion, we have focused on molecular profiling of chemo-naïve tumors from patients with advanced ovarian cancer and optimal tumor residue after PDS, treated with up-front platinum-based chemotherapy. We have described a heterogeneous mutational landscape not only in HGSOC tumor samples with relevance in predicting platinum sensitivity, but also in some uncommon ovarian histotypes. The present study highlighted that the prevalence of driver gene mutations in the different subtypes of advanced ovarian cancer is still matter of debate. A poor prognostic signature (reduced PFI, TTP, OS) was identified in 11% of patients with HGSOC harboring concurrent somatic mutations in two driver actionable genes within a panel of 26 tumor-related genes. In this subgroup of patients, refractory HGSOC was more prevalent than the other patients with one mutated gene or without mutations in genes on the panel. The entire coding regions of *TP53* were sequenced, providing additional value to our study. *TP53*-GOF mutations were a hallmark of platinum-sensitive/intermediate ovarian cancer in patients with HGSOC. Our results shed light on the heterogeneity of ovarian tumors, suggesting multiple routes to the tumorigenic process and the challenges in developing distinct mutational targets. Considering the small group of patients in which a poor prognostic signature was identified, the results for PFI, TTP, and OS should be viewed as hypothesis generating. Considering the current use of PARP inhibitors, other limits are represented by the incomplete knowledge of the *BRCA1/2* mutation status for all the remaining patients, also at the somatic level, and the unknown contribution of potential alterations in genes of the HR pathway. Finally, our study is exploratory and further studies are required for validation to refine the clinical value of the results. However, our data may contribute to improving our understanding of complex and heterogeneous ovarian disease with the final goal of developing specific therapeutic strategies to improve benefits to and the survival of patients.

## Figures and Tables

**Table 1:** Clinico-pathologic characteristics of patients with advanced ovarian cancer, including HGSOC.

Characteristics	All, n = 79	HGSOC, n = 64
Age		
Median (range)	56.3 (31–81.4)	57.0 (31.0–81.4)
CA-125 at diagnosis U/mL		
Median (range)	855.3 (8.8–13100.0)	832.8 (37.9–13100.0)
Tumor Histology		
Serous	64 (81.0)	64 (100.0)
Endometrioid	5 (6.3)	0 (0.0)
Mixed	3 (3.8)	0 (0.0)
Undifferentiated	2 (2.5)	0 (0.0)
Mucinous	1 (1.3)	0 (0.0)
Transitional	1 (1.3)	0 (0.0)
Clear cells	1 (1.3)	0 (0.0)
Unclassified	2 (2.5)	0 (0.0)
FIGO Stage		
IIIB	3 (3.8)	1 (1.6)
IIIC	58 (73.4)	50 (78.1)
IV	18 (22.8)	13 (20.3)
Tumor Grade ^a^		
G2	19 (24.0)	18 (28.1)
G3	60 (76.0)	46 (71.9)
RD at PDS		
0	40 (50.6)	33 (51.6)
<1 cm	39 (49.4)	31 (48.4)
Lymph Node involvement		
Negative	15 (19.0)	10 (15.6)
Positive	42 (53.2)	34 (53.1)
Unknown	22 (27.8)	20 (31.2)
Treatment		
Carboplatin-Paclitaxel	56 (70.9)	45 (70.3)
Carboplatin-PDL	11 (13.9)	9 (14.1)
Carboplatin	5 (6.3)	5 (7.8)
Other ^b^	7 (8.9)	5 (7.8)
PFI ^c^, months		
Median (range)	8.8 (0.0–87.8)	8.4 (0.0–87.8)
TTR ^c^, months		
Median (range)	14.5 (4.6–93.6)	14.5 (5.4–93.6)
Recurrence	70 (88.6)	56 (87.5)
OS, months		
Median (range)	47.7 (8.3–190.4)	48.2 (13.5–190.4)
Deaths	55 (69.6)	42 (65.6)
Platinum sensitivity ^c^		
Refractory	7 (8.9)	3 (4.7)
Resistant	14 (17.7)	13 (20.3)
Intermediate	20 (25.3)	19 (29.7)
Sensitive	36 (45.6)	28 (43.7)

^a^ One patient with tumor grade G2–G3 was included in grade G3. ^b^ Other platinum-based treatments; ^c^ Two patients (one with HGSOC), were not evaluated due to loss at follow up. HGSOC: high-grade serous ovarian cancer. CA-125: Cancer Antigen 125; FIGO: Fédération Internationale de Ginécologie et d’Obstetrique; RD: residual disease; PDS: primary debulking surgery; PDL: Pegylated Liposomal Doxorubicin; PFI: platinum-free interval; TTR: time to recurrence; OS: overall survival; “platinum-refractory”: disease recurrence during treatment or within 4 weeks from the end of platinum treatment: “platinum-resistant”: disease recurrence within <6 months from the end of platinum treatment; “intermediately sensitive”: disease recurrence between 6 and 12 months from the end of platinum treatment; “platinum-sensitive”: disease recurrence >12 months from the end of platinum treatment.

**Table 2:** Distribution of somatic mutations (presence vs. absence) detected by NGS according to clinico-pathologic characteristics of patients with HGSOC.

Characteristics	n = 64	*TP53* n (%)	*p*	*KRAS* n (%)	*p*	*FBXW7* n (%)	*p*	*PTEN* n (%)	*p*	*APC* n (%)	*p*	*PIK3CA* n (%)	*p*
Tumor Histology													
Serous	64	49 (76.6)	-	4 (6.2)	-	2 (3.1)	-	2 (3.1)	-	1 (1.6)	-	1 (1.6)	-
FIGO Stage ^a^													
III	51	39 (76.5)	1.000	1 (2.0)	0.102	2 (3.9)	1.000	2 (3.9)	1.000	1 (2.0)	1.000	1 (2.0)	1.000
IV	13	10 (76.9)		2 (15.4)		0 (0.0)		0 (0.0)		0 (0.0)		0 (0.0)	
Tumor Grade													
G2	18	12 (66.7)	0.326	3 (16.7)	0.064	1 (5.5)	0.487	1 (5.5)	0.487	0 (0.0)	1.000	0 (0.0)	1.000
G3	46	37 (80.4)		1 (2.2)		1 (2.2)		1 (2.2)		1 (2.2)		1 (2.2)	
RD at PDS													
0	33	25 (75.7)	1.000	1 (3.0)	0.347	1 (3.0)	1.000	2 (6.1)	0.493	0 (0.0)	0.484	0 (0.0)	0.484
<1 cm	31	24 (77.4)		3 (9.7)		1 (3.2)		0 (0.0)		1 (3.2)		1 (3.2)	
Platinum sensitivity ^b^													
Refractory	3	1 (33.3)	0.017	1 (33.3)	0.202	1 (33.3)	0.019	0 (0.0)	0.660	1 (33.3)	**<0.001**	0 (0.0)	0.502
Resistant	13	12 (92.3)		0 (0.0)		0 (0.0)		1 (7.7)		0 (0.0)		0 (0.0)	
Intermediate	19	18 (94.7)		1 (5.3)		0 (0.0)		0 (0.0)		0 (0.0)		1 (5.3)	
Sensitive	28	19 (67.8)		2 (7.1)		1 (3.6)		1 (3.6)		0 (0.0)		0 (0.0)	

^a^ One patient with IIIB stage was included in IIIC (III vs. IV). ^b^ One patient was not evaluated due to loss at follow up. NGS: Next-generation sequencing; FIGO: Fédération Internationale de Ginécologie et d’Obstetrique; RD: residual disease; PDS: primary debulking surgery. Comparisons were performed using Fisher’s Exact test or Chi-squared test; according to Bonferroni, *p* value < 0.008 was considered significant and was depicted in bold.

**Table 3:** Associations between number of mutated genes and outcomes in patients with HGSOC.

		Median Survival	Univariate			Multivariate ^#^		
	No. of Patients	Time (Months)	HR ^*^	95% CI ^*^	*p* ^*^	HR ^*^	95% CI ^*^	*p* ^*^
**PFI** ^a^								
N. of mutated genes								
0	12	18.5	Ref.	-	-	Ref.	-	-
1	44	8.6	1.23	0.62–2.43	0.559	1.35	0.63–2.87	0.439
>1	7	8.1	2.55	1.10–5.92	**0.029**	3.10	1.13–8.48	**0.028**
**TTR** ^a^								
N. of mutated genes								
0	12	24.2	Ref.	-	-	Ref.	-	-
1	44	14.4	1.18	0.57–2.43	0.656	1.29	0.59–2.85	0.523
>1	7	13.5	2.57	1.07–6.19	**0.035**	3.14	1.13–8.74	**0.028**
**OS**								
N. of mutated genes								
0	12	66.0	Ref.	-	-	Ref.	-	-
1	45	47.7	1.31	0.47–3.64	0.597	1.47	0.51–4.23	0.469
>1	7	34.0	2.58	0.91–7.34	0.076	3.40	1.14–10.12	**0.028**
**PFI** ^a^								
N. of mutated genes								
0 or 1 ^b^	56	9.0	Ref.	-	-	Ref.	-	-
>1	7	8.1	2.17	1.14–4.11	**0.018**	2.44	1.19–4.99	**0.015**
**TTR** ^a^								
N. of mutated genes								
0 or 1 ^b^	56	14.9	Ref.	-	-	Ref.	-	-
>1	7	13.5	2.25	1.19–4.26	**0.012**	2.54	1.27–5.09	**0.008**
**OS**								
N. of mutated genes								
0 or 1 ^b^	57	48.8	Ref.	-	-	Ref.	-	-
>1	7	34.0	2.06	1.19–3.56	**0.009**	2.47	1.50–4.07	**<0.001**

^*^ Estimated through Cox proportional hazard model; ^#^ Adjusted for residual tumor after PDS, FIGO stage and age at diagnosis. ^a^ One patient was not evaluated due to loss at follow up. ^b^ Patients without (0) mutated genes and with only 1 mutated gene within the panel were grouped. Ref.: Reference Category; PFI: platinum free interval; TTR: time to recurrence; OS: overall survival. *p* values <0.05 were considered significant and were depicted in bold.

**Table 4:** Main clinico-pathologic characteristics of patients with HGSOC and number of mutated genes within the panel.

Characteristics	>1	0 or 1	*p*	1	*p*	0	*p*
	n = 7	n = 57	(>1 vs. 0 or 1)	n = 45	(>1 vs. 1)	n = 12	(>1 vs. 0)
Age							
Median (range)	56.3 (48.5–72.1)	56.8 (31.0–81.4)	0.389 ^*^	56.1(31.0–80.3)	0.318 ^*^	58.9 (42.0–81.4)	ns ^*^
FIGO Stage ^a^							
III	6 (85.7)	45 (78.9)		35 (77.8)		10 (83.3)	
IV	1 (14.3)	12 (21.1)		10 (22.2)		2 (16.7)	
Tumor Grade							
G2	3 (42.9)	15 (26.3)	0.391	11 (24.4)	0.369	4 (33.3)	1.000
G3	4 (57.1)	42 (73.7)		34 (75.6)		8 (66.7)	
RD at PDS							
0	4 (57.1)	29 (50.9)	1.000	22 (48.9)	1.000	7 (58.3)	1.000
<1 cm	3 (42.9)	28 (49.1)		23 (51.1)		5 (41.7)	
Lymph Node involvement							
Negative	2 (28.6)	8 (14.0)	0.092	6 (13.3)	0.100	2 (16.7)	0.167
Positive	1 (14.3)	33 (57.9)		26 (57.8)		7 (58.3)	
Unknown	4 (57.1)	16 (28.1)		13 (28.9)		3 (25.0)	
Platinum sensitivity ^b^							
Refractory	2 (28.6)	1 (1.8)	**0.019**	0 (0.0)	**0.004**	1 (8.3)	0.515
Resistant	1 (14.3)	12 (21.0)		10 (22.2)		2 (16.7)	
Intermediate	2 (28.6)	17 (29.8)		15 (33.3)		2 (16.7)	
Sensitive	2 (28.6)	26 (45.6)		19 (42.2)		7 (58.3)	

^*^ Mann-Whitney test; ^a^ One patient with IIIB stage was included in IIIC (III vs. IV). ^a^ One patient with tumor grade G2-G3 was included in grade G3. ^b^ One patient was not evaluated due to loss at follow up. ns: not significant; FIGO: Fédération Internationale de Ginécologie et d’Obstetrique; RD: Residual Disease; PDS: primary debulking surgery. Comparisons were performed between the sub-group with 2 mutated genes (>1) and patients with: 1 mutated gene or not mutated (>1 vs. 0 or 1); patients with 1 mutated gene (>1 vs. 1); patients not mutated (>1 vs. 0). Fisher’s Exact test, Chi-squared test or Mann-Whitney test were used in the analyses; *p* values <0.05 were considered significant and were depicted in bold.

## Supplementary Materials

The following are available online at https://www.mdpi.com/2073-4409/8/6/584/s1, Figure S1: Type of alteration found by NGS in profiled tumors of advanced ovarian cancer (All, n = 79), including HGSOCs (HGSOC, n = 64), Figure S2: Kaplan-Meier survival curves for PFI, TTR and OS in patients with HGSOC and somatic mutations within the 26 cancer-genes panel, Figure S3: Kaplan-Meier survival curves for PFI, TTR and OS in patients with somatic mutations in *TP53* gene, Table S1: Somatic gene alterations in 79 cases of advanced ovarian cancer; Table S2: Associations between number of somatic mutations in *TP53* gene and outcomes in patients with advanced ovarian cancer (All, n = 79), including HGSOCs (HGSOC, n = 64).
