# Supplementary material for: New Challenges in Tumor Mutation Heterogeneity in Advanced Ovarian Cancer by a Targeted Next-Generation Sequencing (NGS) Approach
Source: Cells. 2019 Jun 14;8(6):584. doi: 10.3390/cells8060584 (PMC6627128; doi:10.3390/cells8060584)
Supplement: Supplementary file 1 [file cells-08-00584-s001.zip › cells-519896-supplementary final.pdf]

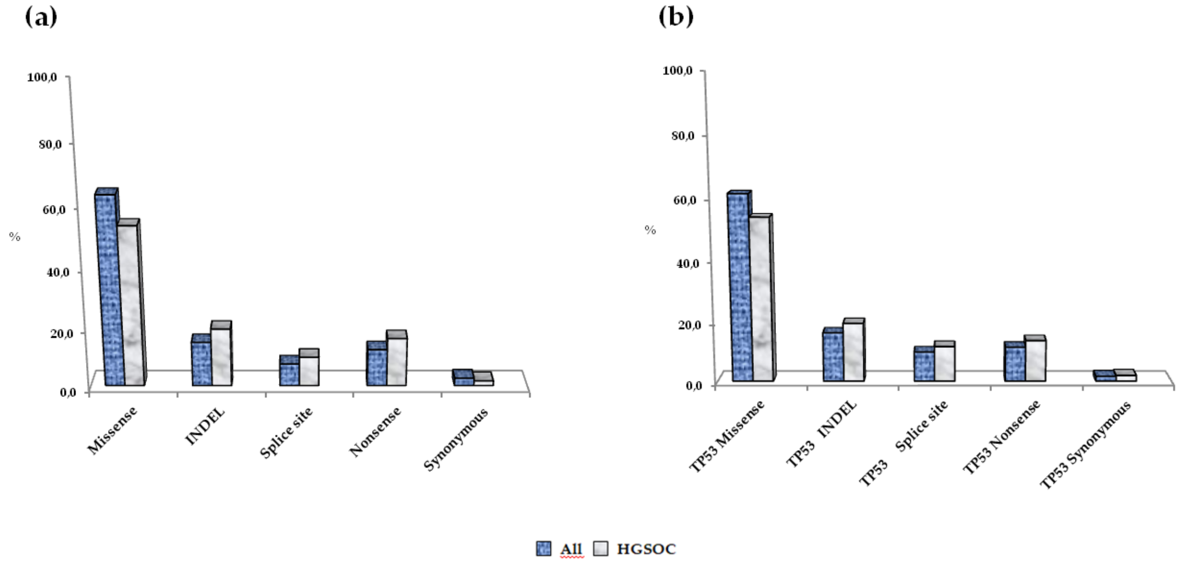

Figure S1. Type of alteration found by NGS in profiled tumors of advanced ovarian cancer (All, n=79), including HGSOCs (HGSOC, n=64) **(a)** Bar graph representation of type of alteration in profiled tumors of advanced ovarian cancer, including HGSOCs **(b)** Bar graph representation of type of *TP53* gene alteration in profiled tumors of advanced ovarian cancer, including HGSOCs. INDEL: insertion or deletion leading to in-frame or frameshift change; HGSOC: high-grade serous ovarian cancer.

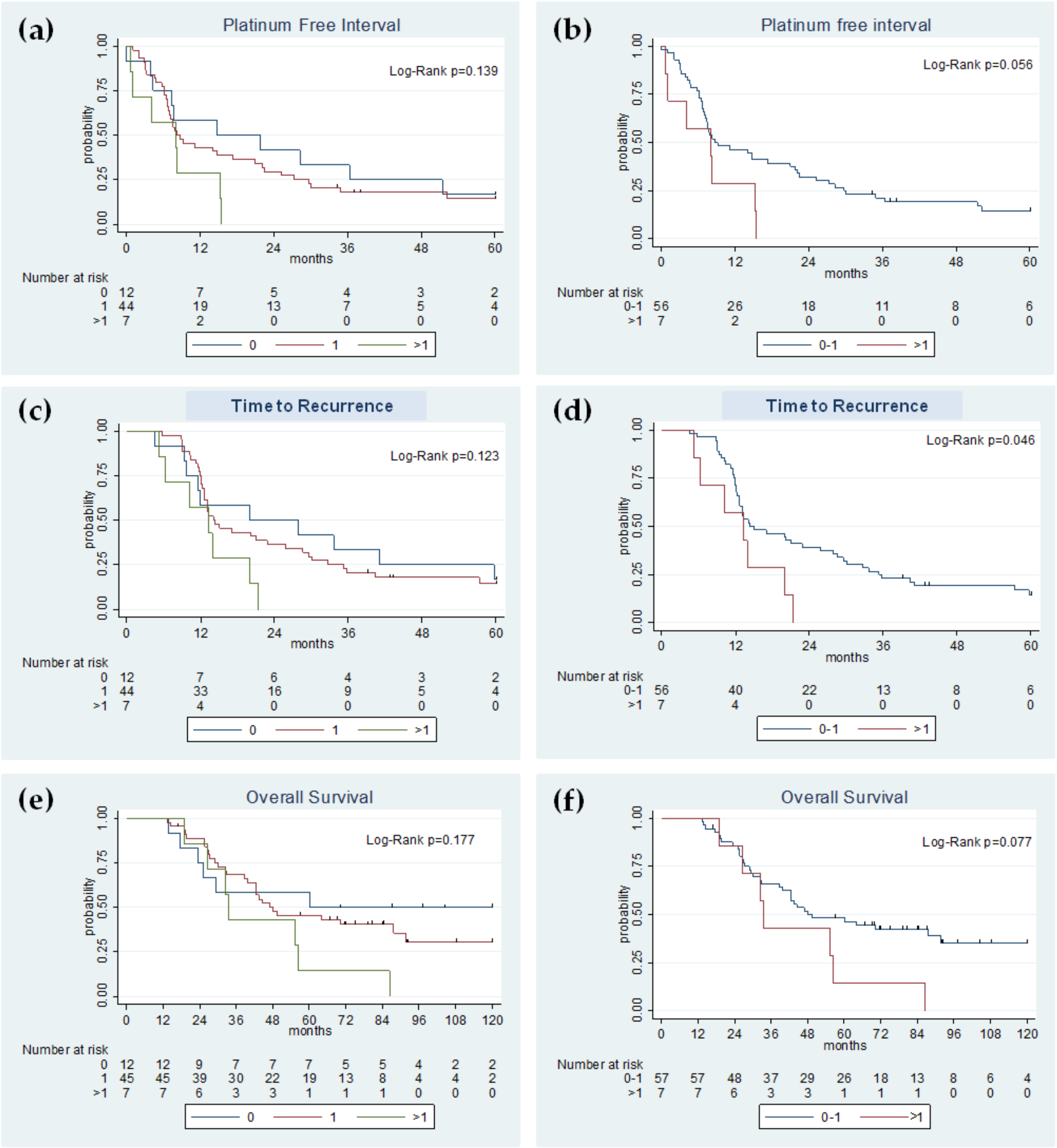

Figure S2. Kaplan-Meier survival curves for PFI, TTR and OS in patients with HGSOC and somatic mutations within the 26 cancer-genes panel. **(a)** PFI curves for patients (n=63) with 2 concurrent somatic mutations in 2 driver genes of the panel (>1, green line), 1 somatic mutation in 1 gene of the panel (1, red line) and without somatic mutations in genes within the panel (0, blue line). **(b)** PFI curves for patients (n=63) with 2 concurrent somatic mutations in 2 driver genes of the panel (>1, red line) and patients with 1 somatic mutation in 1 gene or without somatic mutations in genes within the panel (0-1, blue line). **(c)** TTR curves for patients (n=63) with 2 concurrent somatic mutations in 2 driver genes of the panel (>1, green line), 1 somatic mutation in 1 gene of the panel (1, red line) and without somatic mutations in genes within the panel (0, blue line). **(d)** TTR curves for patients (n=63) with 2 concurrent somatic mutations in 2 driver genes of the panel (>1, red line) and patients with 1 somatic mutation in 1 gene or without somatic mutations in genes within the panel (0-1, blue line). **(e)** OS curves for patients (n=64) with 2 concurrent somatic mutations in 2 driver genes of the panel (>1, green line), 1 somatic mutation in 1 gene of the panel (1, red line) and without somatic mutations in genes within the panel (0, blue line). **(f)** OS curves for patients (n=64) with 2 concurrent somatic mutations in 2 driver genes of the panel (>1, red line) and patients with 1 somatic mutation in 1 gene or without somatic mutations in genes within the panel (0-1, blue line). HGSOC: high-grade serous ovarian cancer; PFI: platinum free interval; TTR: time to recurrence; OS: overall survival.

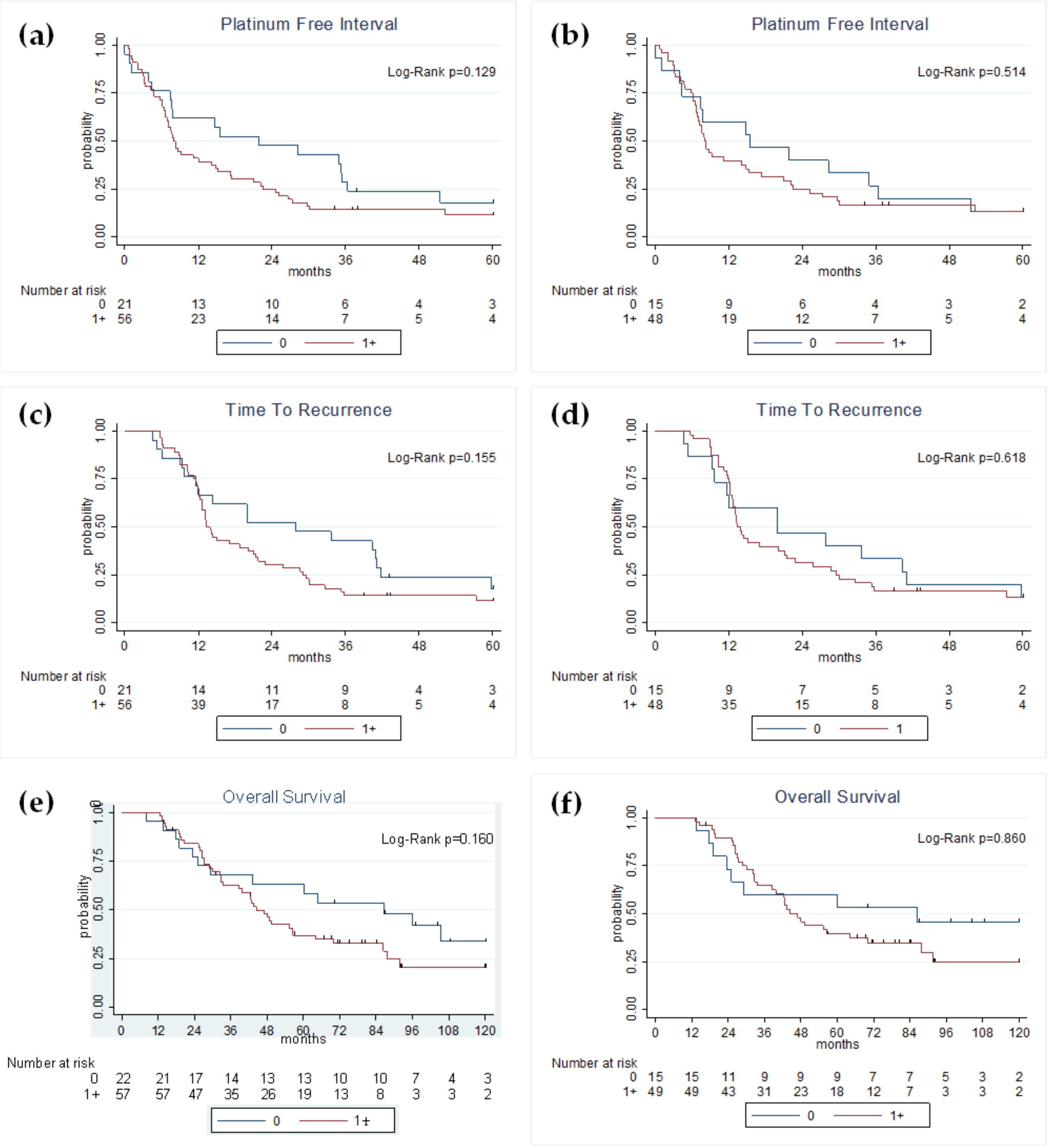

Figure S3. Kaplan-Meier survival curves for PFI, TTR and OS in patients with somatic mutations in *TP53* gene. **(a)** PFI curves for patients with at least 1 somatic mutation in *TP53* (1+, red line) and without somatic mutations in *TP53* (0, blue line), in advanced ovarian cancer (n=77). **(b)** PFI curves for patients with at least 1 somatic mutation in *TP53* (1+, red line) and without somatic mutations in *TP53* (0, blue line), in HGSOC (n=63). **(c)** TTR curves for patients with at least 1 somatic mutation in *TP53* (1+, red line) and without somatic mutations in *TP53* (0, blue line), in advanced ovarian cancer (n=77). **(d)** TTR curves for patients with at least 1 somatic mutation in *TP53* (1+, red line) and without somatic mutations in *TP53* (0, blue line), in HGSOC (n=63). **(e)** OS curves for patients with at least 1 somatic mutation in *TP53* (1+, red line) and without somatic mutations in *TP53* (0, blue line), in advanced ovarian cancer (n=79). **(f)** OS curves for patients with 2 concurrent somatic mutations in 2 driver genes of the panel (>1, red line) and patients with at least 1 somatic mutation in *TP53* (1+, red line) and without somatic mutations in *TP53* (0, blue line), in HGSOC (n=64). HGSOC: high-grade serous ovarian cancer; PFI: platinum free interval; TTR: time to recurrence; OS: overall survival.
