# Supplementary material for: New Challenges in Tumor Mutation Heterogeneity in Advanced Ovarian Cancer by a Targeted Next-Generation Sequencing (NGS) Approach
Source: Cells. 2019 Jun 14;8(6):584. doi: 10.3390/cells8060584 (PMC6627128; doi:10.3390/cells8060584)
Supplement: Supplementary file 1 [file cells-08-00584-s001.zip › Table S2-1.pdf]

Table S2. Associations between number of somatic mutations in *TP53* gene and outcomes in patients with advanced ovarian cancer (All, n=79), including HGSOCs (HGSOC, n=64).

			Median survival	Univariate			Multivariate[#]		
		n. of patients	time (months)	HR[*]	95% CI[*]	*p*[*]	HR[*]	95% CI[*]	*p*[*]
	**All (n=79)**								
**PFI**[a]									
	N. of *TP53* mutations								
0		21	22.1	Ref.	-	-	Ref.	-	-
≥1		56	8.2	1.53	0.89-2.65	0.125	1.70	0.91-3.21	0.098
**TTR**[a]									
	N. of *TP53* mutations								
0		21	28.2	Ref.	-	-	Ref.	-	-
≥1		56	13.8	1.50	0.85-2.63	0.160	1.65	0.86-3.15	0.128
**OS**									
	N. of *TP53* mutations								
0		22	68.3	Ref.	-	-	Ref.	-	-
≥1		57	44.0	1.58	0.81-3.08	0.180	1.65	0.76-3.54	0.203
	**HGSOC (n=64)**								
**PFI**[b]									
	N. of *TP53* mutations								
0		15	15.7	Ref.	-	-	Ref.	-	-
≥1		48	8.2	1.23	0.68-2.24	0.495	1.46	0.74-2.89	0.277
**TTR**[b]									
	N. of *TP53* mutations								
0		15	20.3	Ref.	-	-	Ref.	-	-
≥1		48	13.8	1.17	0.62-2.20	0.619	1.37	0.67-2.79	0.391
**OS**									
	N. of *TP53* mutations								
0		15	71.1	Ref.	-	-	Ref.	-	-
≥1		49	45.1	1.41	0.59-3.35	0.435	1.60	0.65-3.94	0.303

[*]Estimated through Cox proportional hazard model; [#]Adjusted for residual tumor after PDS, FIGO stage and age at diagnosis. [a]Two patients were not evaluated due to loss at follow up. [b]One patient was not evaluated due to loss of follow-up. Ref.: Reference Category; PFI: platinum free interval; TTR: time to recurrence; OS: overall survival.
